# Ultrasound-Guided Central Venous Access With Different Anesthesia Methods in Neonatal Intensive Care Unit

**DOI:** 10.7759/cureus.15753

**Published:** 2021-06-19

**Authors:** Mustafa Okumuş, Adil Umut Zubarioglu

**Affiliations:** 1 Department of Pediatric Surgery, Yeni Yüzyıl University, Faculty of Medicine, Gaziosmanpaşa Hospital, Istanbul, TUR; 2 Department of Pediatrics, Yeni Yüzyıl University, Faculty of Medicine, Gaziosmanpaşa Hospital, Istanbul, TUR

**Keywords:** ultrasound-guided central venous access, newborn, general anesthesia, sedation, internal jugular vein catheterization., central venous access

## Abstract

Background: Ultrasound (US)-guided internal jugular vein (IJV) catheterization in newborns is usually performed in the operating room with general anesthesia. This study aimed to show that US-guided IJV catheterization can be successfully performed with local anesthesia and sedation in newborns.

Methods: The files of newborn patients who underwent US-guided IJV catheterization between May 2017 and May 2020 were examined. Two groups were created according to the type of anesthesia applied during the procedure. The general characteristics of the newborns, the success of the procedure, the number of punctures, and the complication rates in both groups were compared.

Results: A total of 53 newborns were included in this study. Of the 62 procedures, 30 were performed under general anesthesia (group A) and 32 were performed under sedation (group B). Twenty-six (86.6%) of the newborns in group A and 19 (59.3%) in group B were catheterized at the first puncture. The median puncture numbers in groups A and B were 1 (1-3) and 1 (1-5), respectively. All of the patients in group A were successfully catheterized (n = 30; 100%), and all but one in group B could be catheterized (n = 32; 96.8%).

Conclusion: No significant differences in complications or procedural success rates were observed between newborns undergoing general anesthesia or sedation. US-guided IJV catheterization can be safely performed with sedation alone.

## Introduction

Central venous access (CVA) is a necessity in critically ill patients. It is used for medical treatment, intravenous fluids and electrolytes, and parenteral nutrition, as well as monitoring vital signs and painless and easy collection of blood samples [1,2]. CVA is a common procedure among pediatric surgeons and is traditionally performed with the landmark method or open surgical cutdown. Since 2002, the National Institute for Clinical Excellence has endorsed ultrasound (US) guidance as to the method of choice for accessing the jugular vein in both adults and children [3]. As such, US-guided CVA has become a widely applied technique, even in newborns.

The improvement of neonatal intensive care conditions and the enhancement of surgical and medical care both have significantly increased the survival rates of newborns. Inevitably, however, the need for central venous catheterization has gradually increased in newborns, and it has become the most frequently performed invasive procedure in the neonatal intensive care unit (NICU) [4,5]. These procedures are usually performed in the operating room with general anesthesia [1,4,6,7]. As a result, newborns who already require medical care are also exposed to the potential risks of general anesthesia.

At our institution, we perform all such procedures in the NICU. Although some of these procedures are performed in patients already receiving mechanical ventilation support, others are conducted in spontaneously breathing patients with local anesthesia and sedation. In sharing our experience on the subject, by comparing the success and complication rates between these two groups, we aimed to show that US-guided internal jugular vein (IJV) catheterization can be successfully performed with local anesthesia and sedation in newborns.

## Materials and methods

After gaining approval from the local ethics committee (Yeni Yüzyıl University Ethical Committee; IRB no: 2020/41), the files of newborn patients who underwent US-guided IJV catheterization between May 2017 and May 2020 were examined retrospectively. Two groups were created according to the type of anesthesia applied during the procedure. One group consisted of patients already on mechanical ventilation support and curarized during the procedure, and the other comprised spontaneously breathing patients sedated with midazolam and fentanyl. Lidocaine was used for local analgesia in both groups. The general characteristics of the newborns, the success of the procedure, the number of punctures, the intervention duration, and the complication rates in both groups were compared.

All US-guided IJV catheterization procedures were conducted at the bedside in the NICU. The venipunctures were performed with the patients under general anesthesia (mechanical ventilation support; rocuronium bromide 0.5 mg/kg) or sedated intravenously with midazolam (0.1-0.2 mg/kg) and fentanyl (1-2mcg/kg), in the supine position, with a roll under the shoulders and the head turned to the contralateral side. Lidocaine (2 mg/kg) was used for local anesthesia. A Siemens IOE323 5- to-12-MHz transducer (P300 Acuson; Siemens, Munich, Germany) was used in all procedures. Adopting an aseptic technique, the evaluation and vascular access were performed with the transducer in a short-axis view and under real-time US guidance. Three or four French Multicath 2 (Vygon, Écouen, France) catheters with a straight-tip nitinol guidewire were used in all patients. The punctures were made with a 21-gauge echogenic needle. The procedure was considered effective when blood was aspirated. Then, the catheter was introduced using the Seldinger technique. The heart rate, respiratory rate, and oxygen saturation were closely monitored throughout the procedure. The location of the catheter tip was confirmed by a chest X-ray. The right IJV was always preferred as the access route in the first procedure, while the left IJV was preferred for use in the secondary procedures; the right IJV was then used again in the tertiary procedures. No intervention was performed in the subclavian or femoral veins. All procedures were performed by the same senior pediatric surgeon.

All statistical analysis was performed using SPSS version 26.0 (Armonk, NY: IBM Corp.). Mean, standard deviation, median, frequency, and ratio values were used to describe the data. The distribution of variables was measured with the Kolmogorov-Smirnov test. An independent-samples t-test and the Mann-Whitney U test were used in the analysis of quantitative data. In the analysis of qualitative data, the chi-squared test was used; when conditions were not met, Fisher’s exact test was used. Statistical significance was set at p < 0.05.

## Results

A total of 53 newborns with a gestational age range of 23-39 weeks and a mean gestational age of 37 ± 0.7 weeks were included in the study. The weight of these newborns during their respective procedures varied between 1023 g and 5570 g, with a mean weight of 2750 ± 816 g. Ultrasound-guided CVA was performed twice in five patients and thrice in two patients; all of these additional procedures, performed at distinctly different times, were considered separate cases from one another. Of the 62 procedures performed in 53 patients, 30 were performed under general anesthesia (group A) and 32 were performed under sedation (group B). No statistically significant difference existed between the general characteristics of these two groups (Table 1). Twenty-six (86.6%) of the newborns in group A and 19 (59.3%) in group B were catheterized at the first puncture. The difference between the two groups was statistically significant (p=0.016). The number of punctures in group A varied between one and three, and the median was one puncture. In group B, the number of punctures varied between one and five, and the median was one puncture. All of the patients in group A were successfully catheterized (n = 30; 100%), and all but one in group B (n = 32; 96.8%). The patient who could not be catheterized also underwent the most puncture attempts (n = 5). Two days after the failed intervention, a catheter was successfully inserted in the left IJV with US guidance on the first puncture; the patient already had peripheral vascular access and a central venous catheter was not urgent. This intervention was not considered a separate case and was not included in the total number. Two small venous hematomas occurred in group B, but no major complications occurred in the study population. No issues related to guide insertion were recorded. When the number of punctures, intervention durations, and complications was compared between the study groups, no statistically significant difference was observed (Table 2). Mechanical ventilation support or open surgical intervention were not required in any patient who underwent sedation alone.

**Table 1:** Patient characteristics

	Group A general anesthesia n = 30	Group B sedation n = 32	p-Value
Birth weight (grams)	2679 ± 838	2630 ± 794	0.812
Gestational age (weeks)	36.2 ± 3.7	36.4 ± 2.9	0.750
Age at the time of the procedure (days)	17.8 ± 12.7	24.7 ± 16.1	0.066
Weight during the procedure (grams)	2798 ± 616	2923 ± 723	0.469

**Table 2:** The success and complication rates

	Group A general anesthesia n = 30	Group B sedation n = 32	p-Value
Success	30/30 (100%)	31/32 (96.8%)	0.329
Success at the first puncture	26/30 (86.6%)	19/32 (59.3%)	0.016
Number of punctures (median)	1 (1-3)	1 (1-5)	0.124
Duration of intervention (minutes)	10.5 (7-21)	11.5 (7-37)	0.061
Complications	0/30	2/32 (6.25%) 2 hematomas	0.164

## Discussion

This study shows that sedation is as sufficient as general anesthesia in newborns for successful US-guided IJV catheterization, with 31 of 32 patients (96.8%) successfully catheterized with sedation alone. Moreover, the one patient who could not be catheterized initially was successfully catheterized under the same conditions two days later. Ultimately, the success rate in this study group was 100%. Although the success rate at the first puncture attempt was statistically different, no significant difference existed between the two groups in overall success and complications.

Although the increased number of punctures in CVA may lead to higher rates of complications, such as hematoma, pneumothorax, and hemothorax [1,8], a prolonged intervention time facilitates heat loss, especially in preterm newborns [9]. Therefore, the goal should be to reduce the number of punctures and shorten the intervention time in central venous catheterizations. Four important factors are believed to directly affect the success rate of central venous catheterizations: patient cooperation, the experience level of the operator, technique, and the suitability of the instruments used [10,11]. All US-guided CVA procedures in this study were performed by the same senior pediatric surgeon who had performed more than 300 procedures. A suitable device and a probe were used for imaging. Considering the difficulty of advancing a J-tip guidewire in narrow vessels, a straight-tip nitinol guidewire was used in all procedures.

Central venous access is particularly challenging in newborns due to the small diameter of their vessels and the proximity of these vessels to adjacent structures [1,10]. Therefore, during US-guided CVA procedures in newborns, the patient should be immobilized, in addition to the need for an experienced operator. Often, the procedures are performed in an operating room by pediatric vascular specialists or pediatric surgeons under general anesthesia. Administering anesthesia to preterm and full-term newborns involves a high risk of morbidity, particularly postoperative respiratory complications such as apnea and the need for mechanical ventilation [12,13]. However, some reports also suggest that the procedure can be successfully performed with only sedation in the NICU [5,14]. We employed midazolam and fentanyl for sedation, but chloral hydrate, phenobarbital, and ketamine are also effective options [14]. However, the most commonly used drug combination is midazolam and fentanyl [15,16]. In this study, no patient required mechanical ventilation during the procedure. Having a neonatologist and pediatric surgeon on hand is typically sufficient for procedure success, and using the operating room is not necessary. Therefore, US-guided CVA performed with sedation in newborns also reduces the procedure time and cost.

The first choices for venous access in newborns are peripheral lines, umbilical catheters, and peripherally inserted central catheters (PICCs). CVA can be considered when these options are not viable (during the study period, an umbilical catheter was used in 134 patients, and a PICC line was used in 66 patients). The traditional landmark method or open surgical cutdown can be used depending on the surgeon’s preference. However, these procedures are characterized by significant complications related to the surgery or the multiple puncture attempts at catheterization of the central vein, such as arterial puncture, pneumothorax, hemothorax, and localized hematoma. The initial catheterization failure rate of the landmark method in pediatric patients has reached up to 60% in some reports [6,7]. Given the currently available evidence, US-guided CVA can be positioned as an important alternative to this approach. The published results show that the overall success rates associated with US-guided CVA are high and the complication rates are low relative to the traditional methods [17-19]. In general, success rates with US-guided CVA vary between 90% and 100% in the literature, while complication rates range between 4% and 22%. Although some authors have argued that US-guided CVA has no advantage over the landmark method and that US guidance is unnecessary [20], others have found that open surgery in CVA is safer and has lower complication rates [21]. In this study, we catheterized 61 of 62 patients (98.3%) and encountered no major complications such as pneumothorax or hemothorax.

The IJV, subclavian vein, brachiocephalic vein, axillary vein, and femoral vein are the access options that can be co-opted for US-guided CVA in children and even newborns. Among these, the first and most commonly used is the IJV, which is easier to puncture [1]. Ultrasound guidance for CVA could facilitate direct identification of arteries, veins, and adjacent structures, improving the safety of the procedure. The vein can be visualized either in the transversal (short-axis view) or longitudinal (long-axis view) plane, depending on the position of the probe [10,22]. The short-axis view is often used because reliance on the long-axis view during IJV puncture necessitates considerable operator experience, and this intervention area is very small in neonates, increasing the chance for failure. At our institution, we similarly use the short-axis view method when treating newborns because it is easy to adopt. Although reports have stated that catheterization of the subclavian and brachiocephalic veins is easier given their diameter in newborns, we prefer to use the IJV for all interventions, including secondary interventions [23-25]. Undoubtedly, the most important reason for this preference is that we encountered no problems with jugular vein patency in pre-procedure evaluations. In case of failure, our first choice would be the brachiocephalic vein, as we use in infants and children.

Notably, as the number of punctures increase, the intervention duration goes up. Also the risk of developing a complication increases. The hematoma and vasospasm that develop due to the increase in the number of punctures complicate the process. In such cases, it is necessary to change the intervention site. Sometimes postponing is even better if the patient is not in an emergency. As it is seen in our single case, success came two days later. We think that while deciding if the procedure failed, one should check the occurrence of hematoma, hypothermia, or the increased need for sedation rather than counting the number of punctures, such as five or seven punctures [5].

One limitation is worth mentioning in this study. The patient groups were configured retrospectively and not randomly. Since one of the groups consisted of already intubated patients, randomization was not possible.

## Conclusions

Ultrasound-guided CVA in the newborn is a particularly challenging intervention that requires training and experience. When it is performed under general anesthesia, the success rate is significantly high in the first puncture, since the patient is completely immobile. It may reduce the complication rates as it reduces the number of punctures. We believe that it may be a more suitable method for beginners. However, no significant difference was found in complications or success rates between performing the procedure under general anesthesia or sedation; as such, it can be performed safely with sedation alone. With appropriate equipment, it can be performed as a bedside procedure with a high success rate and low complication rate in the NICU.
